# Identifying Glycemic Variability in Diabetes Patient Cohorts and Evaluating Disease Outcomes

**DOI:** 10.3390/jcm10071477

**Published:** 2021-04-02

**Authors:** Martin C. Nwadiugwu, Dhundy R. Bastola, Christian Haas, Doug Russell

**Affiliations:** 1 Department of Biomedical Informatics, University of Nebraska at Omaha, Omaha, NE 68182, USA; 2 Department of Information Systems and Quantitative Analysis, University of Nebraska at Omaha, Omaha, NE 68182, USA; christianhaas@unomaha.edu; 3 Think Whole Person Healthcare, Omaha, NE 68106, USA; team.russell@thinkhealthcare.org

**Keywords:** comorbidity, diabetes mellitus, glycemic variability, HbA1c

## Abstract

Glycemic variability (GV) is an obstacle to effective blood glucose control and an autonomous risk factor for diabetes complications. We, therefore, explored sample data of patients with diabetes mellitus who maintained better amplitude of glycemic fluctuations and compared their disease outcomes with groups having poor control. A retrospective study was conducted using electronic data of patients having hemoglobin A1C (HbA1c) values with five recent time points from Think Whole Person Healthcare (TWPH). The control variability grid analysis (CVGA) plot and coefficient of variability (CV) were used to identify and cluster glycemic fluctuation. We selected important variables using LASSO. Chi-Square, Fisher’s exact test, Bonferroni chi-Square adjusted residual analysis, and multivariate Kruskal–Wallis tests were used to evaluate eventual disease outcomes. Patients with very high CV were strongly associated (*p* < 0.05) with disorders of lipoprotein (*p* = 0.0014), fluid, electrolyte, and acid–base balance (*p* = 0.0032), while those with low CV were statistically significant for factors influencing health status such as screening for other disorders (*p* = 0.0137), long-term (current) drug therapy (*p* = 0.0019), and screening for malignant neoplasms (*p* = 0.0072). Reducing glycemic variability may balance alterations in electrolytes and reduce differences in lipid profiles, which may assist in strategies for managing patients with diabetes mellitus.

## 1. Background

The signs and severity of diabetes mellitus differ among people of different ethnic group and countries, and there are currently no cures for the disease [1]. About two-thirds of people with diabetes mellitus are of working age; in 2017, it was globally estimated that about 425 million people are affected, and this number is predicted to rise to 629 million in 2045 [2,3]. Amongst all countries surveyed, the United States had the highest number of people (over 33,000,000) affected with diabetes mellitus [3] with 84 million people considered pre-diabetic, and 10% of people in this category transition to type 2 diabetes annually [4]. It is currently estimated that an average lifetime economic cost of about $85,000 (or more with increasing age) is needed for clinically managing the disease and its associated comorbidities [5]. Moreover, about 50% of all people with the disease are undiagnosed, which further increases the cost and complexities of managing their condition [6,7].

The clinical treatment goal in managing patients with diabetes mellitus is to prevent the onset of associated comorbidities by relieving symptoms and controlling high and low blood glucose episodes upon diagnosis [1]. Variability in the treatment of diabetes mellitus may arise from the presence or absence of comorbidity, which could be further impacted by self-treatment leading to severe consequences and a change in blood glucose status [8]. Studies have given evidence that chronic high blood glucose is a major factor in the pathogenesis of all diabetes-related comorbidities [9]. Apart from high and low blood glucose levels, glucose variability is another associated risk for complications in diabetes mellitus, necessitating therapeutic approaches aimed at avoiding low glycemic episodes and maintaining a balance in glucose levels [9]. Glucose variability (GV) is more studied in type 1 diabetes(T1D) where it has been suggested to be higher than in type 2 diabetes (T2D) [9].

It is pertinent that glucose levels are kept in a tight normal range in diabetic conditions because although medication may lower glucose levels, lack of continuous monitoring results in unacceptable daily variation in the low and high blood glucose range (<70 and >180 mg/dL) respectively [9]. Mathematical measures such as standard deviation (SD), percentage coefficient of variation (%CV), the mean amplitude of glycemic excursions (MAGE), mean absolute glucose change (MAG), mean of daily differences (MODD), continuous overlapping net glycemic action (CONGA), low blood glucose index (LBGI), high blood glucose index (HBGI), average daily risk range (ADRR), etc., have been proposed for measuring intraday or interday (less commonly used) glycemic variability (GV) [9,10,11,12,13]. These metrics have been used to quantify dynamic variability in glucose concentration profiles from continuous glucose monitoring (CGM) sensors [14].

The objective of the study was to find a group of patients with diabetes mellitus from sample electronic health record (EHR) data to identify those that maintained better amplitude of glycemic fluctuations and compare their disease outcomes, i.e., their associations with a particular diagnosis and/or comorbidity with groups having poor glycemic control. The outcome of interest was to find differences in disease outcomes from clusters of patients with varying glycemic control to see whether aggressive care and management could result in better health outcomes and explain differences across the patient population. The term “diabetic complication” used throughout this study was operationally defined as any comorbidities resulting from long-term diabetes mellitus, while “risk for disease” was defined as factors that increase the possibility for the onset of diabetes mellitus. Recommendations from the study will inform and assist in better medical decisions and patient care management.

This study utilized the control variability grid analysis (CVGA)–a statistical approach to identify glycemic variability in electronic medical record of patients with diabetes mellitus. The CVGA is a representation of the minimum/maximum blood glucose values on a graph and is used to visually assess the quality of glycemic control in a population [15]. The CVGA is based on the variability grid analysis (VGA) method described by Breton and Kovatchev [16]. One important function of the VGA is that it can be used to track glycemic variability at the population level [16]. This study was aimed at finding a suitable threshold to distinguish between stable and unstable glycemia from the electronic data of a group of patients with diabetes mellitus, which is vital in comparing patient glycemic fluctuations and performance across groups [15]. Next, the study applied LASSO (least absolute shrinkage and selection operator) for selecting important diagnosis codes and their influence on glucose control. LASSO is a penalized regression method that can be used for estimating comorbidity outcomes because it selects and retains important variables, providing interpretability and transparency within the regression framework [17,18]. To evaluate disease outcomes by patient cohort, the differences in the presence or absence of the selected diagnosis codes were analyzed using chi-Square and Fisher’s exact test, while their association with each patient cohort was examined using Bonferroni chi-Square adjusted residual analysis.

The overarching goal of the study was to (1) identify, cluster, and validate glycemic fluctuations from data of patients with diabetes mellitus and (2) to evaluate eventual disease outcome, identifying beneficial factors. The remaining sections of this paper will highlight the purpose of the study and explain the methods used. Next, a brief overview of the findings will be discussed and the paper will conclude with how subjects with diabetes mellitus may benefit from future improvements and management strategies. 

## 2. Purpose of the Study

The degree of GV depends on an individual’s physiological parameters of glucose–insulin kinetics, insulin secretion, and sensitivity [11]. It is essential to know the GV in fasting plasma glucose within diabetes subjects and between different time points to be able to appropriately prescribe medication, and keep glucose levels within an acceptable range that reduces the risk of hypoglycemia and other associated complications. The quality of glycemic control is accessed by the GV; however, GV estimate is rarely obtained, which necessitates the need to quantify it [12]. Quantifying GV across a patient population will allow for understanding differences in disease outcomes and for deciding appropriate management strategies for patients with normal glycemic levels and those with erroneous control, respectively.

Additionally, there is a need to identify patterns across clusters of patients with diabetes mellitus for an actionable decision-making process that informs further clinical management strategies employed by healthcare professionals. Apart from indicating the quality of glycemic control and antihyperglycemic therapies, GV has been suggested as an independent risk factor for diabetes complications [19,20,21]. Findings from comparing GV across and within groups and in association with diabetes complications and diagnosis codes (ICD-10) will assist in evaluating disease outcomes and providing a possible explanation for patient encounters that kept glycemic levels under tight control with reduced amplitude. We are able to understand how comorbidities, contact with health facilities, medical screening, and non-adherence influences blood glucose fluctuations by using ICD codes and relating them to the GV profiles of patients with diabetes mellitus.

## 3. Methods

This retrospective study used EHR data with no identifiable information from Think Whole Person Healthcare (TWPH)—a large independent primary care center located at Omaha, United States, and serving over 40,000 patients [22]. The TWPH sees patients with chronic conditions who need ongoing care coordination [22]. The medical records for diabetic patients used in this study captured datapoints on diagnosis codes, demography (age, gender, race), number of medications (antidiabetic drug class), and hemoglobin A1c (HbA1c) levels. The patients who had type-2 diabetes were classified as diabetic based on their HbA1c values.

To maintain research integrity and in full compliance with the Health Insurance Portability and Accountability Act (HIPAA), the data were de-identified and the Office of Regulatory Affairs (ORA) at the University of Nebraska Medical Center (UNMC) reviewed the study proposal and determined that it does not constitute human subject research as defined at 45CFR46.102. An Institutional Review Board (IRB) exempt was approved.

## 4. Eligibility Criteria

Only patients with diabetes mellitus (type 2) having glycated HbA1c values with five time points were included from the electronic medical data of 40,000 patients. For patients with more than five time points, only the most recent five records were included. HbA1c is a blood test that tells the physician the average level of blood sugar present in a patient’s blood over a period of three months [23]. It has the advantage of reflecting glycemic control status [24] and may not reflect short-term changes in glucose control [1]. The rationale for choosing the latest five time points of HbA1c values was to evaluate blood glucose fluctuation for a sustained period to have an overview of glycemic control, since five time points of HbA1c is estimated to a timeline of about 15 months.

A modified CVGA was used to calculate between time point variability of a patient’s glucose profile and within-series variability of HbA1c. The CVGA was obtained by making a plot on the X and Y coordinates, representing the minimum and maximum blood glucose levels for each patient at a particular period of time [15,16]. The coefficient of variability (CV) and amplitude of blood glucose fluctuation was visualized at the population level using the variability grid analysis (VGA) plot [16,25]. Next, diagnosis codes were used to identify comorbidities and other explanations for patient encounters in line with the outcome of interest. These data were included as comorbidity indicators according to the International Classification of Diseases, Tenth Revision, and Clinical Modification (ICD-10-CM). Comorbidities and diagnosis codes present in at least 10 percent of the sampled data were included, while those with less than 10% prevalence were excluded leaving a total of 83 diagnosis codes which were further analyzed for feature selection using LASSO.

## 5. Statistical Analysis

GV was measured using the coefficient of variation (CV), which is an estimation of the standard deviation (SD) of GV divided by the mean blood glucose value. It is an easy-to-calculate and a reliable marker to assess the amplitude of GV [9]. The formula for CV can be represented as shown below in Equation (1), where Rj is the actual blood glucose value at time j and R is the blood glucose value [11].

(1) $$\mathrm{CV}=\frac{\mathrm{SD}}{\mathrm{Mean}};\;\mathrm{SD}=\surd \sum \frac{\left(\mathrm{Rj}-R\right)2}{n-1}$$

Apart from accessing amplitude, studies have suggested that CV is an efficient marker of GV when combined with other metrics of glycemic control [26]. CV can be used to compare fluctuations between stable and unstable diabetes subjects by using its upper limits in healthy individuals as a threshold for comparison between two groups to reveal differences in subgroups exhibiting stable (%CV ≤ maximum CV) and unstable (%CV ≥ maximum CV) glycemic fluctuations [9]. %CV was used to find a threshold to distinguish between stable and unstable GV based on the recommendation that the relative blood glucose deviation must be less than 20% [27,28] and suggestions that the ideal target for %CV should be less than 33% [20]. In this study, %CV was calculated from the data of patients with diabetes mellitus based on their SD and mean HbAIc values and this was used to cluster patients into four cohorts from best to worst, namely: best CV ≤ 10%, intermediate CV from 10% to 20%, high CV from 20% to 30%, very high CV > 30% according to CV classification by Faria et al. [29]. The term “glucose control” was operationally defined using the %CV range. Patients having diabetes mellitus with %CV range between 0 and 10% were operationally defined as having the best glucose control, those having %CV greater than 10% but less than 20% were defined as having good control, while those within the %CV range greater than 20% but less than 30% were defined as having intermediate-to-poor glucose control, and those above 30% were classified as having poor/erroneous glucose control.

Important diagnosis codes were selected using LASSO with %CV as the target variable. LASSO is a penalized regression tool which adds a regularization term (sum of absolute values of coefficient estimates) to the least squares loss function to estimate coefficients [17,18]. The penalty or amount of regularization added to the least squares loss function is determined by multiplying the regularization term with lambda (λ)–a model hyperparameter, as seen in Equations (2)–(4). Apart from being a penalized regression method that models estimation, LASSO also simultaneously computes how the model influences the outcome variable. In this study, LASSO was applied as a method for feature selection; cross-validation using the default number of folds (10 folds) was implemented to obtain the optimal lambda value having the minimal cross-validation mean standard error [30].

(2) $$\lambda \overset{p}{\underset{j=1}{\sum }}\left|\beta j\right|\;\mathrm{Penalty}$$

(3) $$\overset{n}{\underset{i=1}{\sum }}{\left(yi-\;\beta o-\overset{p}{\underset{j=1}{\sum }}\beta jxij\right)}^{2}\;\mathrm{Ordinary}\;\mathrm{least}\;\mathrm{square}\;\mathrm{estimates}\;$$

(4) $$\overset{n}{\underset{i=1}{\sum }}{\left(yi-\;\beta o-\overset{p}{\underset{j=1}{\sum }}\beta jxij\right)}^{2}\;+\;\;\lambda \overset{p}{\underset{j=1}{\sum }}\left|\beta j\right|=RSS+\;\lambda \overset{p}{\underset{j=1}{\sum }}\left|\beta j\right|\;\mathrm{LASSO}$$

In our study, the beta coefficients selected by LASSO represented the independent influencers of %CV and were used for comparing differences in disease outcomes across patient cohorts using the Pearson chi-Square test for homogeneity as well as the Fisher’s exact test given the non-parametric distribution of the data. The Pearson chi-Square test (χ^2^) in Equation (2) is a non-parametric tool used to analyze differences between two factors with nominal levels [31,32]. The Fisher exact test was applied where the assumptions for chi-Square test were not met, i.e., where the expected value for the chi-Square test statistics (χ^2^) were below 5 [31]. In the analysis, the factors (i.e., the dependent variable) were the presence or absence of comorbidity as indicated by the diagnosis codes, while the independent categorical variables were the four patient cohorts.

(5) $$\chi^{2}=\underset{i}{\sum }\underset{j}{\sum }{\left(\frac{fij-\;eij}{eij}\right)}^{2}$$

A probability value less than 0.05 was used as the criteria for statistical significance in all statistical analysis carried out. Although the Pearson chi-Square test tells whether there is a significant difference in the presence or absence of comorbidity across the patient cohort, it is important to know where this significance comes from. Therefore, the Bonferroni chi-Square adjusted residual analysis—a post hoc test, was used to test for cell/cohort significance after a statistically significant chi-Square test was obtained [32]. The test was done to find associations of specific comorbidity and ICD code with each of the cohorts for statistical inference. Multivariate Kruskal–Wallis (MKW) test was used to determine whether differences in age and number of medications taken (continuous dependent variables) vary by cohort level (categorical independent variable). All data were analyzed using R software version 3.6.0 and IBM SPSS Statistics version 26.0.0 on Windows.

## 6. Results

A total of 3333 diabetes patient’s data consisting of 1795 (55.03%) males and 1467 (44.97%) females met the eligibility criteria; only 3262 records had information on diagnosis codes. The data consisted of 2583 Whites, 129 African Americans, 27 Asians, 7 American Indian or Alaska Native, other Pacific Islander 1, undefined 501, and unreported 14. The baseline statistics reporting the patient’s %CV, age, number of medications, and antidiabetic drug class in each cohort can be found in Table 1.

### 6.1. Visualizing Glycemic Control with VGA Plot

**Figure 1:**
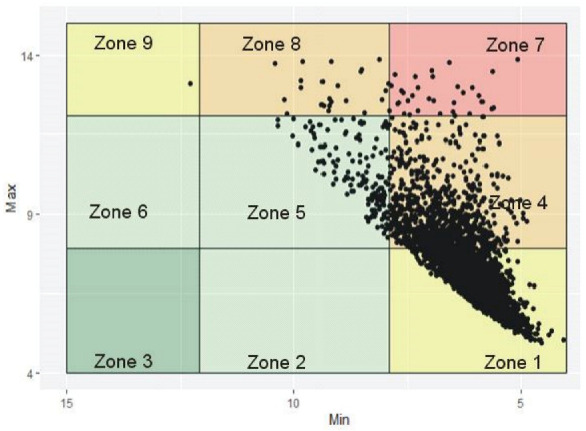
Modified variability grid analysis (VGA) plot: Zone 1: shows optimal control with points <5 showing deviations into hypoglycemia, Zone 4: control of GV amplitude, Zone 5: departures into hyperglycemia, Zone 7: poor glucose control, Zone 8: poor control of hyperglycemia, Zone 9: excessive neutralization of hypoglycemia.

Figure 1 shows the glycemic control of all patients using the modified CVGA plot [16]. The modified CVGA plot is a minimum/maximum plot of the HbA1c values for a patient observed over an arbitrary period. The plot is split into zones; the minimum HbA1c value is plotted on the x-axis, while the maximum HbA1c value is plotted on the y-axis and the difference represents the amplitude observed for a patient. Zone 1 is the area of optimal glycemic control with points less than 5 showing deviations into hypoglycemia. Majority of the patients within this zone have HbA1c values that were never above 7.89, where 6.5 is considered the strict target value for people with diabetes [33]. HbA1c recommended guideline of a range between 7.5 and 6.5 in type 2 diabetes patients was reported by Pfeiffer and Klein [34]. Zone 4 has a HbA1c range from 12.08 to 4.0 and shows suboptimal control of blood glucose variations. Zone 5 represents departures into hyperglycemia while zone 7 represents an area of poor glucose control with the greatest amplitude (below 7.89 and above 12.08 HbA1c values). Zone 8 represents an area of poor hyperglycemic control, while Zone 9 is an area of excessive neutralization of hypoglycemia. The explanation of data points in each zone comes from the VGA model created by Breton and Kovatchev [16] and discussed by Kovatchev and Cobelli [11].

### 6.2. Distinguishing between Stable and Unstable GV Using %CV

**Figure 2:**
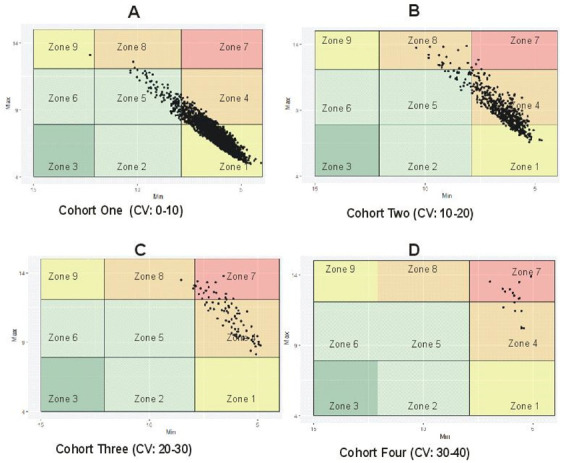
Coefficient of variability (CV): four cohorts of minimum/maximum glycemic variability (GV) plot based on %CV range. (**A**) Patients in the hypoglycemic and hyperglycemic range with low %CV, (**B**) intermediate %CV, (**C**) high %CV, (**D**) very high %CV.

The threshold for stable and unstable GV was determined using %CV for blood glucose fluctuations. The %CV have been described as a reliable marker for assessing the amplitude of glycemic fluctuations, because it adjusts the mean glucose value [9]. Subjects having %CV less than 10 were considered the most stable and were clustered as the best cohort with low variability, while those with %CV greater than 30% were the most unstable cohort with very high variability as seen in Figure 2 [27,28,29]. In our sample data, the percentage of patients exhibiting low, intermediate, high, and very high %CVs were 80.5, 16.4, 2.49, and 0.51 in cohorts 1, 2, 3, and 4, respectively. In brief, the %CV was used as a range to distinguish between stable and unstable GV irrespective of the subjects being in the hypoglycemic or hyperglycemic range. Figure 2A shows that most patients had good glycemic control (80.5%), while patients having poor/erroneous control were in the minority and had the least data points (0.51%) as seen in Figure 2D.

### 6.3. Selecting Important Diagnosis Codes and Influencers of %CV

**Figure 3:**
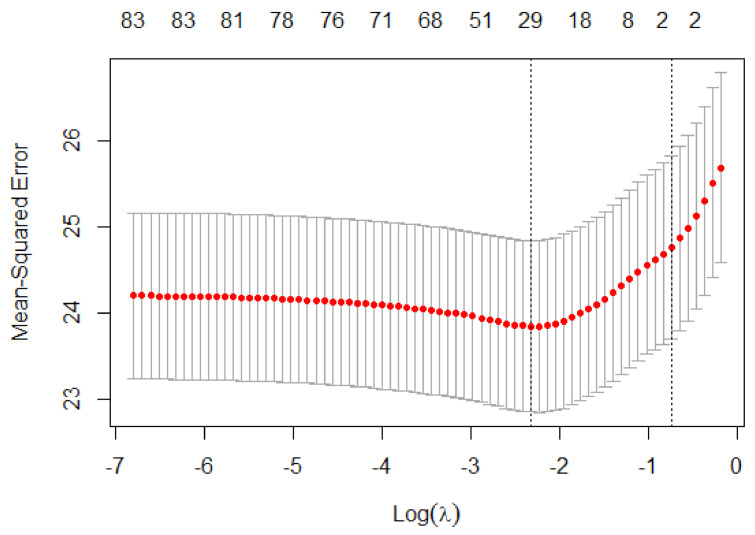
Least absolute shrinkage and selection operator (LASSO): cross-validation plot.

**Figure 4:**
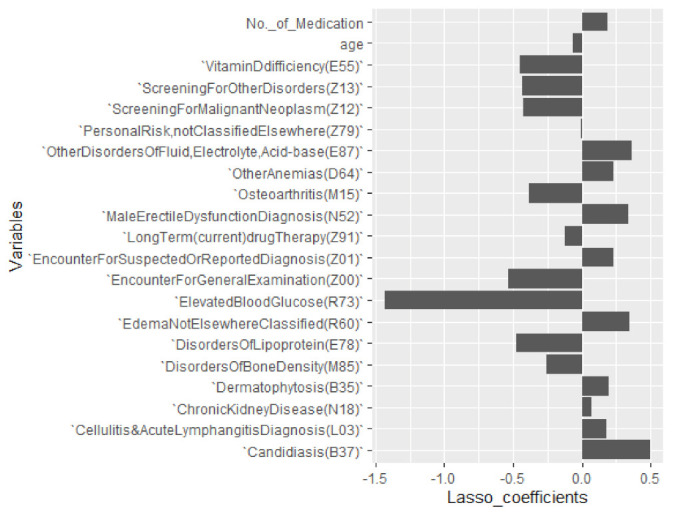
LASSO: a plot showing important variables selected and their coefficients with respect to %CV.

Out of 83 diagnosis codes, LASSO regression (R^2^ = 0.1062483, λ = 0.1051296) with %CV as the target variable selected 22 important influencers of GV including age and number of medications along with their beta estimates (coefficient) while shrinking other variables to zero as can be seen in Figure 3 and Figure 4. The beta estimates describe the effects of the respective diagnosis codes, age, and number of medications by antidiabetic drug class on the %CV. Figure 3 shows the cross-validation plot and lambda with the best minimal standard error. Among the LASSO-selected variables, 15 were comorbidity indicators, while 7 (31.8%) were ICD-10-CM diagnosis codes (Z00-Z99), which are associated with factors influencing health status, contact with health services, and non-adherence to medical treatment. The diagnosis codes, coefficients, and ICD-10 explanations are reported in Table 2.

LASSO regression showed that candidiasis (B37); other anemias (D64); other disorders of fluid, electrolyte, acid–base balance (E87); edema, not elsewhere classified (R60); and male erectile dysfunction (N52) were the most positive %CV influencers, while elevated blood glucose (R73), disorders of lipoprotein (E78), and osteoarthritis (M15) were negative influencers. Within our study population, a patient with R73 has a decrease of approximately −1.43 in %CV, while a patient with E87 has approximately 0.36 increase in %CV with other variables kept constant. Encounters for general examination (Z00) and screening for neoplasms (Z12) were the two main factors applicable to non-adherence to medical treatment that were negatively associated with %CV. The coefficients for LASSO regression showing the relative contribution of each variable to %CV can be found in Table 2.

### 6.4. Differences and Association of Comorbidity by Patient Cohort

From Table 3, elevated blood glucose (*p* = 0.0009); edema (*p* = 0.0206); male erectile dysfunction (*p* = 0.0324); osteoarthritis (*p* = 0.0051); vitamin D deficiency (*p* = 0.0003); disorders of lipoprotein (*p* = 0.0019); bone density (*p* = 0.0031); fluid, electrolyte, acid–base balance (*p* = 0.0009) as well as cellulitis and acute lymphangitis (*p* = 0.0051) significantly varied across patient cohorts, which indicates a difference in their association with %CV. The presence of other variables (Z79, Z01, Z51, N18, B35, D64) were not significantly different across the four patient cohorts in this study population (*p* > 0.05).

In the Bonferroni chi-square adjusted residual analysis as seen in Appendix A, the presence of E78 (*p* = 0.0014) was only significantly associated with cohort 4. E87 was statistically significant in cohort 4 (0.0322) and cohort 2 (0.0360) compared to cohort 1 and 3. The association of disorders of osteoarthritis (M15), bone density (M85), and male erectile dysfunction (N52) were statistically significant in cohort 1 (0.0054, 0.0109, and 0.3055, respectively) compared to the other cohorts. The association of cellulitis and acute lymphangitis (L03) (*p* = 0.0047), edema (R60) (*p* = 0.0355), and candidiasis (B37) (*p* = 0.0005) were significant in cohort 1 and cohort 2 (*p* = 0.0036, 0.0157, and 0.0036), respectively, while vitamin D deficiency (E55) was only significantly associated with cohort 1 (*p* = 0.0017) and cohort 3 (*p* = 0.0313).

The association of factors (Z00–Z99) influencing health status, contact with health services, and non-adherence to medical treatment varied significantly by cohort level. Screening for other disorders (Z13) was associated with only cohort 1 (*p* = 0.0137), while screening for personal risk not classified elsewhere (Z91) was associated with cohort 3 (*p* = 0.0478) and cohort 1 (*p* = 0.0019) but not cohort 4; screening for neoplasms (Z12) was only associated with cohort 1 (*p* = 0.0072) and cohort 2 (*p* = 0.0304) as seen in Appendix A.

Given the data also had continuous dependent variables (age and number of medication), the multivariate Kruskal–Wallis test was used to determine whether distribution of these diagnosis variables were statistically significant (*p* < 0.05) across the patient cohort. The pairwise comparison of %CV and age in cohort 3 and cohort 1 (*p* < 0.000) as well as cohort 2 and cohort 1 (*p* < 0.000) were statistically significant as seen in Table 4; while the pairwise comparison of %CV and number of medications was only statistically significant in cohort 1–cohort 2 (*p* < 0.000). The mean age for patients in cohort 1, 2, and 3 were 69.64, 66.64, and 62.36, respectively, indicating that patients with lower %CV were slightly older within this population. Overall, our data were mainly comprised of older patients and on average, patients in cohort 4 took more combination of drugs, as the mean number of medications taken by patients from cohort 1 to 4 were 0.646, 0.886, 0.778, and 1.188, respectively.

## 7. Discussion

The frequency of hypoglycemia and the mean blood glucose driven by degree and period of hyperglycemia, are two parameters that govern the optimization and stability of diabetes control; and GV is a barrier to attaining this stability [35]. In the modified VGA plot in Figure 1, the zone with optimal glucose control (zone 1) showed tight normal stability with only minor deviations into hypoglycemia, while the zone with erroneous blood glucose control (Zone 7) had the highest instability with frequent episodes of hypoglycemia and hyperglycemia. This instability represents the amplitude of glycemic control, which is a standard assessment of GV [11]. Although Dai et al. [36] reported that HbA1c variability is not a robust predictor of poor glycemic control in the older person, it is vital to note that departures toward hypoglycemia and hyperglycemia having the same amplitude vary in order of importance and severity [11]. A unit rise in HbA1c values toward hyperglycemia does not equal a unit decline toward hypoglycemia, which is more clinically important for optimizing glucose control. Moreover, the risk for hyperglycemia is clinically independent from that of hypoglycemia [25]. Apart from amplitude, the timing of glycemic fluctuations is also considered clinically relevant (e.g., in closed-loop control of diabetes) as GV has been suggested as a process in time [11]. This study did not consider the speed of change from one state to another due to the limitation of the data. However, this could be possible on data with real-time reaction to glucose fluctuations and GV estimates [11].

A high coefficient of variability is a measure of GV that may cause harmful effects. GV is independent of hypoglycemia but associated with poor glycemic control and heightened severity of diabetes-related comorbidities [37,38]. GV has been associated with increased protein kinase C-β (PKC) which promotes oxidative stress [37]; and atypical PKC conserved in the liver has been reported to promote lipid and metabolic abnormalities in people with type 2 diabetes [39]. From our study, patients with very high %CV were significantly associated with disorders of lipoprotein and disorders of fluid, electrolyte, and acid–base balance. Patients having diabetes mellitus with erroneous glycemic control are susceptible to dyslipidemia [40], and it has been reported that the risk of contracting several diseases among diabetics is affected by differences in lipid and electrolyte profiles [41]. Although the risk for major cardiovascular outcome (MACE) was not directly significant in the study, a reduction in cholesterol has been strongly touted as a means of lowering the risk for MACE, even in patients with unusual lipid profiles [40]. Lipid changes during 8 weeks of low caloric diet in a trial study by Valsesia et al. [42] have been used to predict insulin-resistant patients. Apart from that, it has been reported that metformin is associated with atherogenic lipid markers such as high- and low-density lipoprotein cholesterol, total cholesterol, etc., and is suggested to be more effective in persons with reduced atherogenic lipid markers [43]. It is however unclear how effective metformin was in cohort 4, given that it was one of the few antidiabetic medications taken by the patients in that cohort; however, data on lipid profiles were not readily available.

A unique strength of this study is the comparison and link between glucose fluctuations and disease outcomes. Analysis showed that patients who had their personal risks classified and were screened for factors influencing health status, including those that have had contact with healthcare services, were more likely to have adequate glycemic control. This may be due to a corrective non-adherence to medical treatment and management regimen, even though the patients may be around the hyperglycemic range and have other existing comorbidities. The findings were in agreement with the study on HbA1c variability and the risk of poor glycemic control among older adults by Dai et al. [36], who reported that frequent specialist visits may indicate the presence of complex multi-morbidity, but reduces the risk of poor glycemic control due to intensive monitoring. Our study showed that patients who had the lowest %CV, some of whom were in the hyperglycemic range, were significantly associated with factors influencing health status such as screening and classification of personal risk and contact with health services (Z00–Z99) despite being significantly associated with disorders of osteoarthritis (M15), bone density (M85), male erectile dysfunction (N52), edema (R60), candidiasis (B37), vitamin D deficiency (E55), cellulitis and acute lymphangitis (L03). While the duration of compliance to medical regimen is unknown, patients with increasing %CV within our population data were less likely to be classified, and therefore it was difficult to decipher whether they followed the prescribed course of treatment. Proper screening, classification of personal risk not otherwise known, and engagement with healthcare services will help in patient compliance with prescribed treatment and management strategies that will probably keep additional complications and diabetes-associated inflammation in check [44].

Factors influencing health status and non-adherence to treatment were negative influencers of %CV as seen in our result reported in Table 2 and Figure 4. Adopting the penalized regression, a patient associated with Z00 and Z12 diagnosis codes within our sampled population for instance has an approximate −0.537 and −0.423 respective decrease in %CV with other variables kept constant. In other words, patients positively associated with diagnosis codes influencing health status had a higher likelihood of having low glycemic fluctuation. Healthcare providers may benefit from adopting this method for further classifying diabetes patients and studying them to reveal patterns that may assist in optimizing care and management strategies for the population they serve.

Additional factors associated with glucose fluctuations were age and number of antidiabetic medications taken. Patients who had very high/erroneous glycemic fluctuation took on average more combinations of drugs. Polypharmacy, which is the use of multiple medications, has been reported to be more prevalent in older adults with diabetes [45] and is associated with adverse drug events, non-adherence to treatment, and drug–disease interaction [45,46]. Apart from polypharmacy, age-related changes and diabetes-related diseases increase the difficulty of managing older persons with diabetes [7,45]. From our study, patients in cohort 1 had the highest mean age (*µ* = 69.64) and on average took less combinations of drugs (*µ* = 0.646), while those in cohort 4 took on average more drugs (*µ* = 1.188) and had a decreased mean age (*µ* = 62.36) compared to the other cohorts. While the number of antidiabetic medications taken was a positive influencer of glycemic fluctuation in our study, age was negatively correlated with %CV. Furthermore, despite the fact that Kasim et al. [47] reported that old age is associated with diabetes, our results with respect to our sample data indicated otherwise, possibly reflecting the synopsis by Kalyani and Egan [48], who posited that although older persons are vulnerable to alterations in glucose metabolism and diabetes-related complications, abnormal glucose metabolism is not an essential component of aging. This probably explains the negative correlation between age and stable glycemic fluctuations.

Overall, it is important to establish that while race was not an important variable with respect to %CV from our analysis, over 79% of our data sample were Whites, which could potentially limit conclusions for non-White patients due to the respective small sample size. Furthermore, it is vital to keep in mind that, while HbA1c is one of the few measures of glycemic control and an influencer of diabetes-related outcomes, there are instances where its use may not be appropriate. For example, HbA1c has been found to vary significantly during different seasons, being lower in warmer months and higher in colder temperatures [49]. It has also been reported to be unreliable in people with variant homozygous hemoglobin such as HbC or HbS than in people with heterozygous variants HbAC or HbAS [24]. It was reported to be falsely elevated in transfused patients due to the amount of glucose composition of the storage vector, in patients with lead poisoning, in people using opioid and salicylate, and in alcoholics [50]. It has also been found to be lowered with the treatment of hemolytic anemia, end-stage renal disease, splenomegaly, and in conditions that shorten the life cycle of red blood cells and reduce erythrocyte exposure to glucose [51,52]. Therefore, healthcare practitioners should consider alternative measures to validate HbA1c test and to question results in these instances.

Generally, a 1% change in HbA1c values corresponds approximately with a 30 mg/dL change in mean glucose levels; so, a more recent change in glucose levels will have a greater influence on HbA1c values than preceding changes, because HbA1c measures the weighted mean of glycemic levels over a three-month period and may not be suitable for estimating per diem fluctuations [50]. The implication is that a patient might have an overall normal glycemic control from HbA1c test even though there could have been preceding changes in glucose levels that may not have been captured which represents another limitation. It is important to note that every index of glycemic control has a limitation; however, the advantage of the HbA1c test is that it is a convenient and good metric for estimating GV and does not require fasting or timed samples. In general, while patients with diabetes mellitus within the four cohorts of our study were linked to a particular diagnosis, this study does not imply a causal relationship between them but rather was carried out to investigate differences in outcomes within the context of a sampled population.

## 8. Conclusions

This study showed that GV is an obstacle to effective blood glucose stability and that the link between glucose fluctuations and risk for disease outcome is minimized with proper screening and engagement with healthcare services for classification of personal risk not otherwise known, as patients with this practice have a higher likelihood of having lower amplitude of glycemic fluctuation. Healthcare providers may benefit from adopting a penalized regression method for further classifying diabetes patients and studying them to reveal patterns in a bid to optimize care and reduce the risk for future disease outcomes.

While age was an important factor in understanding stable glycemic fluctuation, it was not an essential positive influencer for controlling it. Reducing glycemic variability could lower lipid profiles and reduce differences in electrolyte alterations, which may assist in intervention strategies for managing patients with diabetes mellitus. Future prospective and randomized controlled studies are needed to investigate and validate treatments and practices targeting a reduction in glycemic variability.

## Figures and Tables

**Table 1:** Antidiabetic drug class and baseline statistics.

Statistics of %CV, Age, and Medication							
			%CV		Age	No. of Medications	
Number of Patients		Valid	3262		3262	3262	
		Missing	0		0	0	
Mean			6.90		74.36	0.69	
Std. Error of Mean			0.09		0.2	0.022	
Std. Deviation			5.07		13.052	1.271	
Minimum			0.72		21	0	
Maximum			44.51		107	9	
Antidiabetic drug class for control of glucose for each cohort							
Antidiabetic drug class							
Cohort 1	Cohort 2			Cohort 3			Cohort 4
Metformin, Insulins, Sulfonylurea, Insulins, Thiazolidinediones(TZDs), Dipeptidyl peptidase 4 (DPP IV) inhibitors, Combination products, glucagon-like peptide1 (GLP) agonist, DGLT V inhibitors	Metformin, Sulfonylurea, Insulins, GLP agonist, DPP IV inhibitors, Combination Products, TZDs, DGLT V inhibitors			Sulfonylurea, GLP agonist, Metformin, DPP IV inhibitors			Combination products, Insulin, Metformin

**Table 2:** Coefficients for diagnosis codes and International Classification of Disease (ICD)-10 explanations.

Diagnostic Codes and ICD 10 Explanation	Coefficients
Elevated Blood Glucose (R73)	**−1.426739684**
Encounter for General Examination (Z00)	**−0.537150731**
Disorders of Lipoprotein (E78)	**−0.478726309**
Vitamin D Deficiency (E55)	**−0.451607901**
Screening for Other Disorders (Z13)	**−0.428622075**
Screening for Malignant Neoplasm (Z12)	**−0.423327639**
Osteoarthritis (M15)	**−0.383649241**
Disorders of Bone Density (M85)	**−0.259501973**
Long-Term (current) Drug Therapy (Z91)	**−0.124074972**
Age	**−0.063355789**
Personal Risk, Not Classified Elsewhere (Z79)	**−0.001640932**
Chronic Kidney Disease (N18)	0.074005080
Cellulitis and Acute Lymphangitis Diagnosis (L03)	0.181943299
No. of Medications by Antidiabetic Drug Class	0.189978300
Dermatophytosis (B35)	0.192212136
Other Anemias (D64)	0.227729951
Encounter for Suspected or Reported Diagnosis (Z01)	0.229726767
Male Erectile Dysfunction Diagnosis (N52)	0.336851533
Edema Not Elsewhere Classified (R60)	0.349784394
Other Disorders of Fluid, Electrolyte, Acid–base (E87)	0.361342201
Candidiasis (B37)	0.495985527
(Intercept)	12.46255677

Negative coefficients are highlighted.

**Table 3:** Chi-Square and Fisher’s exact test for cohort differences in the presence of comorbidity.

Diagnosis	Χ^2^	df	Asymptotic Significance (Two Sided)	Exact Significance (Two Sided)	ICD 10 Explanation
Z12	11.557	3	**0.0090**		Screening for malignant neoplasm
Z91	16.221	3	**0.0010**		Personal risk, not classified elsewhere
R60	9.7755	3	**0.0205**		Edema, not elsewhere classified
Z00		3		9.29 × $10^{-6}$	Encounter for general examination
R73		3		0.0009	Elevated blood glucose
E78		3		0.0019	Disorders of lipoprotein
M15		3		0.0052	Osteoarthritis
E55		3		0.0003	Vitamin D deficiency
Z13		3		0.0108	Screening for other disorders
M85		3		0.0030	Disorders of bone density
L03		3		0.0050	Cellulitis and acute lymphangitis
N52		3		0.0324	Male erectile dysfunction
E87		3		0.0009	Other disorders of fluid, electrolyte, acid–base
Z79	5.0793	3	0.1661		Long term (current) drug therapy
Z01	2.8959	3	0.4080		Encounter for suspected or reported diagnosis
Z51		3		0.5930	Encounter for other outer, medical care
N18		3		0.5002	Chronic kidney disease
B35		3		0.2162	Dermatophytosis
D64		3		0.2373	Other anemias

Significant *p*-values are highlighted.

**Table 4:** Pairwise comparisons of %CV, number of medications, and age.

Pairwise Comparisons of %CV and Age					
Sample 1–Sample 2	Test Statistic	Std. Error	Std. Test Statistic	Significance	Adjusted Significance
cohort 3–cohort 4	−57.841	257.569	−0.225	0.8220	1.0000
cohort 3–cohort 2	280.031	112.346	2.493	** 0.0130 **	0.0760
cohort 3–cohort 1	537.612	106.203	5.062	** 0.0000 **	0.0000
cohort 4–cohort 2	222.190	238.909	0.930	0.3520	1.0000
cohort 4–cohort 1	479.771	236.082	2.032	** 0.0420 **	0.2530
cohort 2–cohort 1	257.581	44.887	5.738	**0.0000**	0.0000
** Pairwise Comparisons of %CV and Number of Medications **					
cohort 1–cohort 3	−127.472	88.261	−1.444	0.1490	0.8920
cohort 1–cohort 2	−152.083	37.304	−4.077	** 0.0000 **	0.0000
cohort 1–cohort 4	−224.917	196.198	−1.146	0.2520	1.0000
cohort 3–cohort 2	24.611	93.366	0.264	0.7920	1.0000
cohort 3–cohort 4	−97.445	214.055	−0.455	0.6490	1.0000
cohort 2–cohort 4	−72.833	198.547	−0.367	0.7140	1.0000

Significant *p*-values are highlighted.

## Data Availability

The data presented in this study are available on request from the corresponding author, Dhundy Bastola. The data are not publicly available due to ethical restrictions.

## Appendix A

**Table A1:** Bonferroni chi-square adjusted residual analysis.

Crosstab								ICD-10 Explanations
ICD-10	Cohort 1	Cohort 2	Cohort 3		Cohort 4		Total	Disorders of Lipoprotein
E78	Absent	Count	239	64	10	6	317	
		Expected Count	256.3	51.3	7.9	1.6	317.0	
		% within E78	75.4%	19.6%	3.2%	1.9%	100.0%	
		Adjusted Residual	−2.6	1.7	0.8	3.8		
		*p*-value	0.0762	0.6901	0.9100	**0.0014**		
	Present	Count	2398	466	71	10	2945	
		Expected Count	2380.7	476.7	73.1	14.4	2945.0	
		% within E78	81.4%	15.8%	2.4%	0.3%	100.0%	
		Adjusted Residual	2.6	−1.7	−0.8	−3.8		
		Adj. *p*-value	0.0762	0.6901	0.9100	**0.0014**		
M15	Absent	Count	2342	491	77	16	2927	
		Expected Count	2366.2	473.8	72.7	14.4	2927.0	Osteoarthritis
		% within M15	80.0%	16.8%	2.6%	0.5%	100.0%	
		Adjusted Residual	−3.4	2.7	1.6	1.4		
		Adj. *p*-value	**0.0054**	0.0559	0.8756	1		
	Present	Count	294	37	4	0	335	
		Expected Count	270.8	54.2	8.3	1.6	335.0	
		% within M15	87.8%	11.0%	1.2%	0.0%	100.0%	
		Adjusted Residual	3.4	−2.7	−1.6	−1.4		
		Adj. *p*-value	**0.0054**	0.0559	0.8756	1		
E55	Absent	Count	2118	452	76	14	2660	Vitamin D difficiency
		Expected Count	2150.3	430.6	66.1	13.0	2660.0	
		% within E55	79.6%	17.0%	2.9%	0.5%	100.0%	
		Adjusted Residual	−3.7	2.6	2.9	0.6		
		Adj. *p*-value	**0.0017**	0.0688	**0.0313**	1		
	Present	Count	519	76	5	2	602	
		Expected Count	486.7	97.4	14.9	3.0	602.0	
		% within E55	86.2%	12.6%	0.8%	0.3%	100.0%	
		Adjusted Residual	3.7	−2.6	−2.9	−0.6		
		Adj. *p*-value	**0.0017**	0.0688	**0.0313**	1		
Z13	Absent	Count	1828	395	65	13	2301	Screening for other disorders
		Expected Count	1860.1	372.4	57.1	11.3	2301.0	
		% within Z13	79.4%	17.2%	2.8%	0.6%	100.0%	
		Adjusted Residual	−3.1	2.4	1.9	0.9		
		Adj. *p*-value	**0.0137**	0.1496	0.4183	1		
	Present	Count	809	133	16	3	961	
		Expected Count	776.9	155.6	23.9	4.7	961.0	
		% within Z13	84.2%	13.8%	1.7%	0.3%	100.0%	
		Adjusted Residual	3.1	−2.4	−1.9	−0.9		
		Adj. *p*-value	**0.0137**	0.1496	0.4183	1		
M85	Absent	Count	2346	488	79	16	2929	Disorders of bone density
		Expected Count	2367.8	474.1	72.7	14.4	2929.0	
		% within M85	80.1%	16.7%	2.7%	0.5%	100.0%	
		Adjusted Residual	−3.2	2.2	2.3	1.4		
		*P*-value	**0.0109**	0.2326	0.1586	1		
	Present	Count	291	40	2	0	333	
		Expected Count	269.2	53.9	8.3	1.6	333.0	
		% within M85	87.4%	12.0%	0.6%	0.0%	100.0%	
		Adjusted Residual	3.2	−2.2	−2.3	−1.4		
		Adj. *p*-value	**0.0109**	0.2326	0.1586	1		
Z91	Absent	Count	1592	354	62	11	2019	Personal risk, not classified elsewhere
		Expected Count	1632.2	326.8	50.1	9.9	2019.0	
		% within Z91	78.9%	17.5%	3.1%	0.5%	100.0%	
		Adjusted Residual	−3.7	2.7	2.7	0.6		
		Adj. *p*-value	**0.0019**	0.0621	**0.0478**	1		
	Present	Count	1045	174	19	5	1243	
		Expected Count	1004.8	201.2	30.9	6.1	1243.0	
		% within Z91	84.1%	14.0%	1.5%	0.4%	100.0%	
		Adjusted Residual	3.7	−2.7	−2.7	−0.6		
		Adj. *p*-value	**0.0019**	0.0621	**0.0478**	1		
L03	Absent	Count	2263	421	67	14	2765	Cellulitis and acute lymphangitis
		Expected Count	2235.2	447.6	68.7	13.6	2765.0	
		% within L03	81.8%	15.2%	2.4%	0.5%	100.0%	
		Adjusted Residual	3.4	−3.5	−0.5	0.3		
		Adj. *p*-value	**0.0047**	**0.0036**	0.4828	0.6081		
	Present	Count	374	107	14	2	497	
		Expected Count	401.8	80.4	12.3	2.4	497.0	
		% within L03	75.3%	21.5%	2.8%	0.4%	100.0%	
		Adjusted Residual	−3.4	3.5	0.5	−0.3		
		Adj. *p*-value	**0.0047**	**0.0036**	0.4828	0.6081		
N52	Absent	Count	2311	444	67	12	2834	Male erectile dysfunction
		Expected Count	2291.0	458.7	70.4	13.9	2834.0	
		% within N52	81.5%	15.7%	2.4%	0.4%	100.0%	
		Adjusted Residual	2.6	−2.1	−1.1	−1.4		
		Adj. *p*-value	0.0674	0.3055	0.2088	0.1266		
	Present	Count	326	84	14	4	428	
		Expected Count	346.0	69.3	10.6	2.1	428.0	
		% within N52	76.2%	19.6%	3.3%	0.9%	100.0%	
		Adjusted Residual	−2.6	2.1	1.1	1.4		
		Adj. *p*-value	0.0674	0.3055	0.2088	0.1266		
E87	Absent	Count	2309	438	74	10	2831	Other disorders of fluid, electrolyte, acid−base
		Expected Count	2288.6	458.2	70.3	13.9	2831.0	
		% within E87	81.6%	15.5%	2.6%	0.4%	100.0%	
		Adjusted Residual	2.7	−2.8	1.2	−2.9		
		Adj. *p*-value	0.0584	**0.0360**	0.1749	**0.0322**		
	Present	Count	328	90	7	6	431	
		Expected Count	348.4	69.8	10.7	2.1	431.0	
		% within E87	76.1%	20.9%	1.6%	1.4%	100.0%	
		Adjusted Residual	−2.7	2.8	−1.2	2.9		
		Adj. *p*-value	0.0584	**0.0360**	0.1749	**0.0322**		
R60	Absent	Count	1782	320	55	10	2167	Edema, not elsewhere classified
		Expected Count	1751.8	350.8	53.8	10.6	2167.0	
		% within R60	82.2%	14.8%	2.5%	0.5%	100.0%	
		Adjusted Residual	2.8	−3.1	0.3	−0.3		
		Adj. *p*-value	**0.0355**	**0.0157**	0.6213	0.5908		
	Present	Count	855	208	26	6	1095	
		Expected Count	885.2	177.2	27.2	5.4	1095.0	
		% within R60	78.1%	19.0%	2.4%	0.5%	100.0%	
		Adjusted Residual	−2.8	3.1	−0.3	0.3		
		Adj. *p*-value	**0.0355**	**0.0157**	0.6213	0.5908		
B37	Absent	Count	2347	440	66	14	2867	Candidiasis
		Expected Count	2317.7	464.1	71.2	14.1	2867.0	
		% within B37	81.9%	15.3%	2.3%	0.5%	100.0%	
		Adjusted Residual	4.0	−3.5	−1.8	0.0		
		Adj. *p*-value	**0.0005**	**0.0036**	0.5869	0.7693		
	Present	Count	290	88	15	2	395	
		Expected Count	319.3	63.9	9.8	1.9	395.0	
		% within B37	73.4%	22.3%	3.8%	0.5%	100.0%	
		Adjusted Residual	−4.0	3.5	1.8	0.0		
		Adj. *p*-value	**0.0005**	**0.0036**	0.5869	0.7693		
Z12	Absent	Count	1057	249	38	9	1353	Screening for malignant neoplasm
		Expected Count	1093.8	219.0	33.6	6.6	1353.0	
		% within Z12	78.1%	18.4%	2.8%	0.7%	100.0%	
		Adjusted Residual	−3.3	2.9	1.0	1.2		
		Adj. *p*-value	**0.0072**	**0.0304**	0.2517	0.1834		
	Present	Count	1580	279	43	7	1909	
		Expected Count	1543.2	309.0	47.4	9.4	1909.0	
		% within Z12	82.8%	14.6%	2.3%	0.4%	100.0%	
		Adjusted Residual	3.3	−2.9	−1.0	−1.2		
		Adj. *p*-value	**0.0072**	**0.0304**	0.2517	0.1834		

Significant *p*-values are highlighted.
